# Co-producing digital mental health interventions: A systematic review

**DOI:** 10.1177/20552076241239172

**Published:** 2024-04-25

**Authors:** Rebecca Brotherdale, Katherine Berry, Alison Branitsky, Sandra Bucci

**Affiliations:** 1 Division of Psychology & Mental Health, School of Health Sciences, Faculty of Biology, Medicine and Health, Manchester Academic Health Sciences Centre, Manchester, UK; 2 9022 Greater Manchester Mental Health NHS Foundation Trust, Manchester, UK

**Keywords:** Co-production, involvement, digital mental health, mHealth, mental health

## Abstract

**Objective:**

Smartphone apps (apps) are widely recognised as promising tools for improving access to mental healthcare. However, a key challenge is the development of digital interventions that are acceptable to end users. Co-production with providers and stakeholders is increasingly positioned as the gold standard for improving uptake, engagement, and healthcare outcomes. Nevertheless, clear guidance around the process of co-production is lacking. The objectives of this review were to: (i) present an overview of the methods and approaches to co-production when designing, producing, and evaluating digital mental health interventions; and (ii) explore the barriers and facilitators affecting co-production in this context.

**Methods:**

A pre-registered (CRD42023414007) systematic review was completed in accordance with The Preferred Reporting Items for Systematic reviews and Meta-Analyses guidelines. Five databases were searched. A co-produced bespoke quality appraisal tool was developed with an expert by experience to assess the quality of the co-production methods and approaches. A narrative synthesis was conducted.

**Results:**

Twenty-six studies across 24 digital mental health interventions met inclusion criteria. App interventions were rarely co-produced with end users throughout all stages of design, development, and evaluation. Co-producing digital mental health interventions added value by creating culturally sensitive and acceptable interventions. Reported challenges included resource issues exacerbated by the digital nature of the intervention, variability across stakeholder suggestions, and power imbalances between stakeholders and researchers.

**Conclusions:**

Variation in approaches to co-producing digital mental health interventions is evident, with inconsistencies between stakeholder groups involved, stage of involvement, stakeholders’ roles and methods employed.

## Introduction

A global deficit in access to mental health treatment, specifically psychological support^1^ has led to the development, evaluation, and/or deployment of digital mental health interventions (DMHIs), including software applications (apps). A DMHI is defined as an intervention delivering information, support, or therapy for mental health conditions through an electronic medium with the aim of treating, alleviating, or managing mental health difficulties.^2^ DMHIs have demonstrated efficacy, supported by meta-analytic evidence for a range of mental health difficulties, including anxiety^3^ and depression.^4,5^ DMHIs are increasingly regarded as viable alternatives to augment and expand the delivery of mental healthcare.^4,6^

With the widespread availability of smartphones, apps are increasingly being used to address the shortage of access to psychological interventions. Although evidence supports the use of apps for the delivery of DMHIs,^7^ app use over more lengthy periods of time is not sustained.^8^ Misalignment between researchers’ aims and clinical users’ needs has been shown to be a major contributing factor to attrition^9^; specifically, lack of attention to individual user characteristics and poor app design.^10^ Additionally, apps require users to be intrinsically motivated, as users typically engage with apps in their own time.^11^ Therefore, key to continued engagement is understanding end users’ needs and preferences.^12^ In this study, the term ‘end user’ is used to refer to the person who uses or is intended to use the DMHI for mental health support.

Participatory methods provide one means of ensuring that apps are designed to better meet users’ needs and therefore promote longer term engagement. The health literature contains various participatory methods that involve different approaches for collaboration between researchers and stakeholders.^13^ The terminology for these methods is not always consistent, with a multitude of terms used (often interchangeably), including: patient and public involvement (PPI), co-production, co-design and co-creation.^14^ In the absence of a single agreed term, the term ‘co-production’ is used throughout this review. Despite inconsistency in terms, a common principle underlines all methods of co-production: ‘Nothing about us, without us’,^15^^,p.1^ with an underlying ethos of developing *with* and not *for* users,^16^ by placing end users at the heart of the process.

Co-production has a sound theoretical basis and is grounded in self-determination theory, whereby autonomy and relatedness increase the likelihood of behaviour change.^17^ Evidence indicates the indispensability of co-production in developing interventions to enhance outcomes. In a comprehensive review of co-production in mental health more generally, commissioned by the mental health charity, Mind, Slay and Stephens^18^ examined pertinent themes related to well-being, social connectedness, stigma, and the mitigation of acute mental health service utilisation following co-producing services and interventions. Notably, their findings underscored the impact co-production had on improved functioning, characterised by heightened autonomy, competence and relatedness – a congruence with the tenets of self-determination theory.^17^

The importance of co-produced research is also increasingly recognised by national agendas, including the UK's National Institute for Health and Care Research.^18^ Co-production is deemed to be particularly important as it enables the inclusion of minoritized groups and the accommodation of cultural needs by its ability to address mental health inequalities.^19^ It is even possible for researchers to prioritise engaging under-representative groups and ensuring that their voices are embedded throughout decision making processes.^20^

Despite the growing recognition of the importance of co-producing DMHIs with people who experience mental health difficulties,^21^ few DMHIs have involved co-production processes.^22^ Additionally, those DMHIs that have been co-produced are often limited to involvement in only the early and/or final stage of research design/intervention development or delivery,^23,24^ which is contradictory to the core principles of co-production.^25^ Consequently, many mental health apps are publicly accessible from app stores and have many monthly active users,^26^ but lack sufficient evidence regarding their design, development, and evaluation.^27^ One possible barrier in involving users in app design is that there is no standardised guidance on how to involve stakeholders in this process. There are several frameworks for involving users in research more generally, but none focus specifically on involving users in the design of DMHIs more specifically. Involving users in the design of DMHIs can bring additional complexity owing to the diverse and multitude of stakeholders required, including researchers, service users (or ‘end users’), health professionals and app developers.^28^

In terms of user involvement in research frameworks more generally, the NIHR INVOLVE produced guidance around co-producing research^25^ describing five key principles: (a) the sharing of power; (b) inclusion of diverse perspectives and skills; (c) respect and value the knowledge of those working together; (d) reciprocity; and (e) relationship building and maintenance. Additionally, the UK Design Council's double diamond method advises four main phases where involvement can take place; discover (identify the problem), design (define the intervention), develop (develop potential solutions) and deliver (testing). Another tool is the Involvement Matrix, co-produced by Smits et al.,^29^ which defines five main roles within co-production (the listener, co-thinker, advisor, partner and decision-maker). The roles reflect the degree of stakeholder involvement, ranging from passive recipient of information (listeners) to active contributors of options (co-thinkers) and advice (advisors), to equal collaborations (partners) and finally, the highest level, the decision makers. In a systematic review of 22 studies, Veldmeijer et al.^30^ explored the extent to which stakeholders were involved in mental healthcare through design, including DMHIs. The review found most studies involved end users at a ‘co-thinker’ level within the matrix framework developed by Smits et al.,^29^ with no single study involving end users at the highest level as ‘decision maker’. These conclusions were supported by another review of 433 studies by Baines et al.^31^ who explored co-production within digital health innovation, implementation, and evaluation, and found whilst co-production is recognised as essential, it is rarely practised. Whilst these reviews concern co-production, they did not describe the methods and approaches to facilitate co-production, nor explore the barriers and added value to implementing co-production, which would be clinically useful for guiding decisions when co-producing DMHIs. Furthermore, the studies were not limited to the delivery of DMHIs through apps, despite the widespread availability of apps,^32^ and increasing popularity of this platform for mental health treatment.^33^

Some individual studies have described the way in which they have used co-production methods in the development and delivery of DMHIs. However, to our knowledge, there are no published reviews that systematically aggregate the methods and approaches employed. Therefore, our aim is to systematically review the literature to map out the methods of, and approaches to, co-production when designing, producing, and evaluating apps aimed at supporting individuals with mental health difficulties. Notably, to the best of our knowledge, no previous review has focused on apps, despite their rapid scalability and stakeholders’ concern regarding the lack of scientific evidence. We aim to offer an in-depth exploration by synthesising the methods used in co-production of mental health apps and offer recommendations for improving the co-production process to maximise acceptability and engagement with DMHIs. The objectives of this study are to: (i) describe the methods and approaches to co-production that have been used when designing, producing, and evaluating DMHIs delivered via apps; and (ii) explore the barriers and facilitators affecting co-production with DMHIs delivered via apps.

## Methods

The systematic review was conducted and reported in accordance with the Preferred Reporting Items for Systematic Reviews and Meta-Analyses (PRISMA) guidance.^34^ The review protocol was developed in advance and registered with the International Prospective Register of Systematic Reviews of the National Institute for Health Research (PROSPERO CRD42023414007). As this is a mixed studies systematic review, the seven standard systemic review steps for mixed studies reviews have been followed^35^: (i) specifying the review question; (ii) defining eligibility criteria; (iii) applying an extensive search strategy; (iv) identifying potentially relevant studies through rigorous screening (by lead reviewer and an independent reviewer); (v) selecting relevant studies based on full text; (vi) data extraction and study quality appraisal (using the bespoke co-produced quality tool); and (vii) synthesising the data from the included studies.

### Search strategy and identification of studies

Relevant studies were identified through systematic search of five electronic databases: MEDLINE, PsycInfo, Embase, CINAHL plus and Web of Science. Databases were searched in June 2023, from inception to present day. A comprehensive search strategy was developed, with a wide variety of key search terms and was performed by primary author (RU). The PICOS framework^35^ supported the development of the search strategy. Search terms were also informed by title and abstracts of key papers, including review papers.^30,31^ Search terms were categorised into: (a) mental health difficulties, (b) mobile applications and (c) co-production. Free text and Medical Subject Headings (MeSH) terms were used for each database. Search terms within each category were combined with Boolean operator ‘OR’ and the three categories were combined with Boolean operator ‘AND’. Pilot searches were undertaken to help generate the final search terms (see Table 1). Reference lists were browsed for any additional relevant studies and citation chasing was undertaken^36^ to further supplement the search.

**Table 1.** Search terms by category across all databases.

	Database (and platform)	PsycInfo (OVID), MEDLINE (OVID), Embase (OVID), CINAHL plus (EBSCOhost) and Web of Science (Clarivate)
No.	Search Categories	Search Terms
#1	‘Mental health difficulties’	Mental illness* OR mental health OR depressi* OR anxi* OR psycho* OR schizo* OR affective disorder* OR bipolar OR obsessive-compulsive disorder* OR post-traumatic stress disorder* OR eating disorder* OR personality disorder* OR attention deficit with hyperactivity OR autis* OR self-harm* OR suicid* OR dementia OR alzheimer* OR substance abuse* OR sleep disorder*
#2	‘Mobile applications’	Mobile application* OR software application* OR mobile phone* OR cell phone* OR smartphone OR mobile health OR mHealth OR automated
#3	Co-production	Co-produc* OR coproduc* OR co-design* OR codesign* OR co-creat* OR cocreat* OR collaborat* OR participatory design* OR user-led OR user cent*red OR patient and public involvement OR PPI
#4	Final search	#1 AND #2 AND #3

### Study selection

**Figure 1.**
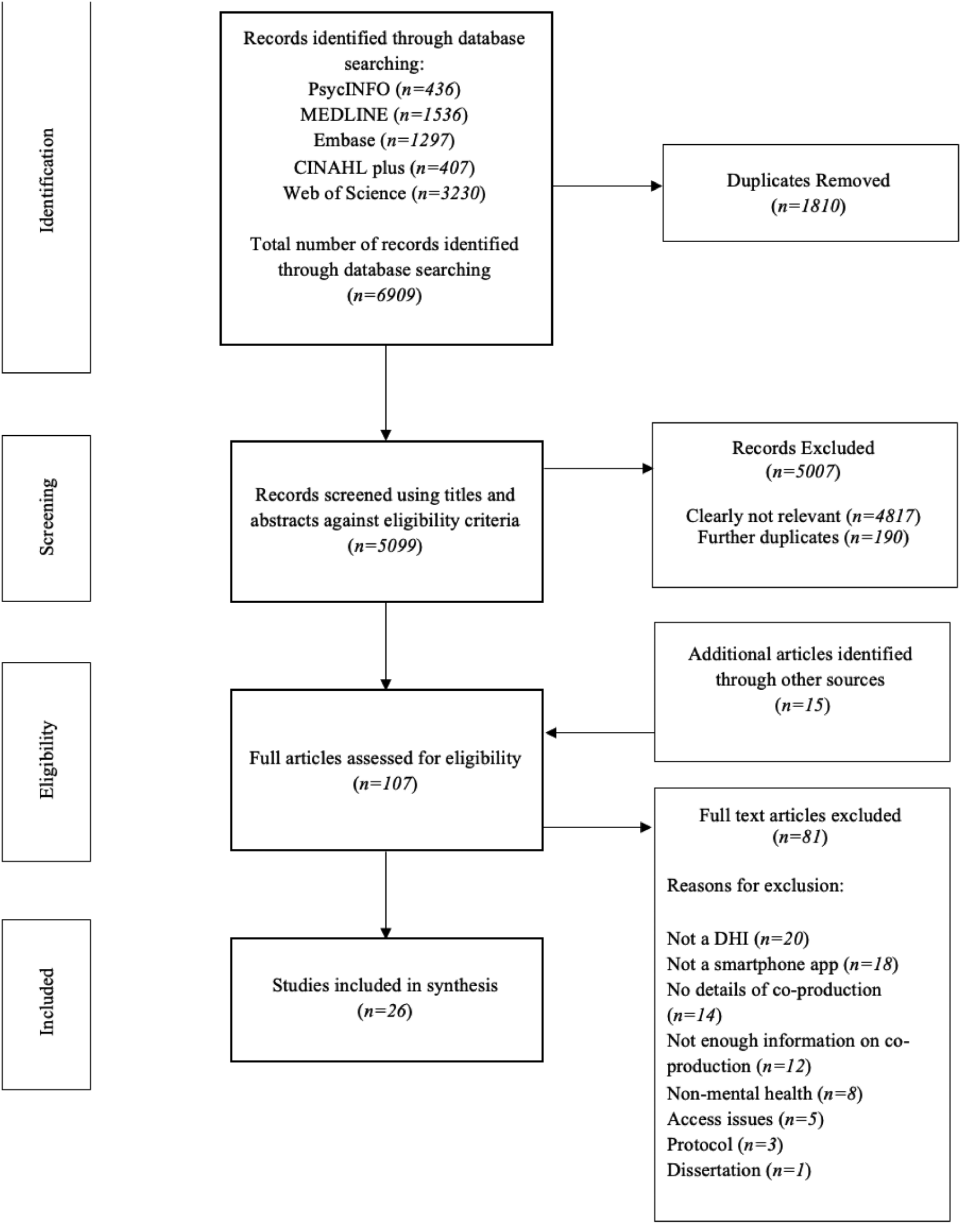
PRISMA flow diagram of systematic search.

Study selection and exclusion processes are outlined in Figure 1. After removing duplicates, article titles and abstracts were screened against the eligibility criteria by author RU. If eligibility was unclear, full text articles were obtained, reviewed in accordance with eligibility criteria and discussed with the wider research team. A second independent rater (SR) screened 15% of titles and abstracts, with any disagreements resolved with the research team. Following this, 15% of the full text articles were also screened by the second rater (SR) to assess reliability of study selection. There was substantial agreement (93%) between raters. Identified discrepancies were resolved by consensus within authors. Disagreements at full text stage were due to whether the app classified as a DMHI targeting mental health difficulties or emotional well-being, and whether there was enough information regarding the co-production process.

Inclusion criteria were: (a) stakeholder involvement (i.e. service users, mental health professionals); (b) studies explicitly describing stakeholder involvement in the design, development and/or evaluation; (c) studies describing a DMHI delivered by a smartphone app designed specifically to target mental health problems; and (d) published in peer review journals, either a qualitative or mixed methods design. DMHIs were defined using the definition from the Hollis et al. (2017, p. 475) review: ‘DMHIs as: information, support and therapy for mental health conditions delivered through an electronic medium with the aim of treating, alleviating, or managing symptoms’. Exclusion criteria were : (a) non-human participants or no details around stakeholders; (b) studies with insufficient information around user-centred design; whereby the co-production process if not explicitly outlined, i.e. detailed co-production happened but does not describe involvement; (c) studies not focused on delivering a DMHI aimed at targeting mental health problems (e.g. focused on general wellbeing); and (d) studies not written or translated into the English language, within the grey literature or full texts not available.

### Data analysis

Data was extracted using a structured form that was developed and piloted on a small number of papers. Extracted data included: stakeholder characteristics, DMHI characteristics, co-production approach, characteristics (method and outcome of co-production) and reported barriers and facilitators to co-production. Author RU extracted the data from the included papers and a second independent of the research team also extracted the same data from 20% of papers to check for consistency. Any extraction disagreements were resolved within the wider research team.

Due to the absence of an established system for rating co-production in the context of DMHIs, and following the ethos of co-production, a bespoke quality rating method based on the Centre for Reviews and Dissemination guidance for conducting systematic reviews (CRD, 2009) was co-produced with an expert by experience (see Supplementary material). Developing the tool entailed five iterative steps: (a) collaborative consultation to explore the purpose of the tool and discuss roles and expectations; (b) collaboratively reviewing the literature, including amalgamating existing key frameworks (NIHR INVOLVE UK Standards for Public Research^18^; the 4pi National Involvement Standards^37^; the UK Design Council's double diamond method (2005); and the Involvement Matrix^29^ to synthesis the key phases and determine areas for quality assessing; (c) developing and testing the prototype tool; (d) refining and formalising the tool within the supervisory team; and (e) testing the final tool. Whilst there are several participatory research frameworks, they all report a similar series of sequential phases. The bespoke tool placed emphasis on the steps taken by the researcher to ensure identification and representation of the population, equality in decision making, and the process of involvement. Studies were not excluded based on quality if they addressed the focus of the review. Moreover, the process of quality assessment ensured rich and meaningful insights from the data were not lost. Thus, studies were not assigned a numerical value in the interest of this being an appraisal of each criterion to better describe the quality of the co-production methods of the studies included, and in accordance with Siddaway et al. (2019) who recommends avoiding summary scores. To establish a reliable rating system, 20% of articles were independently rated by an independent second reviewer with areas of discrepancy identified and disagreements resolved through discussion within the wider team. The quality appraisal results have been integrated throughout the results section given the review is focused on appraising the studies.

A narrative synthesis of the evidence was conducted, which involved presenting the characteristics, findings, and quality appraisal alongside the reported barriers and added value of co-production for each study within tables to enable a within-study synthesis. A cross-study synthesis was then conducted whereby the differences between characteristics and quality appraisal ratings of the studies were reviewed and integrated. This supported the ability to aggregate and synthesise the methods and approaches to co-production with mental health interventions delivered via apps, and the barriers and added value to co-production. ENTREQ checklist was used for transparency.^38^

## Results

### Search results

Initial database search yielded 6906 records. After duplicates were removed, 5099 records were screened by title and abstract. The remaining 107 articles were reviewed for full text screening. Twenty-six papers detailing 24 DMHIs were identified for inclusion in the final synthesis. Included studies were published between 2013 and 2023.

### Study characteristics

The 26 studies are summarised in Table 2. Three studies from one author reported on the same DMHI; but each paper represented a different phase of the co-production process.^59–61^ Therefore, these three papers were combined, totalling 24 studies included for synthesis. Nearly half of the studies (*n* = 10) were conducted in either UK or USA. Studies targeted a range of mental health conditions: psychosis (*n* = 8); depression (*n* = 4); suicidal ideation and crisis support (*n* = 3); mental health but not otherwise specified (*n* = 3); anxiety (*n* = 2); and borderline personality disorder, serious mental illness, relational difficulties and depression and anxiety (1 study each). Not all studies detailed a therapeutic modality; however, 10 studies reported delivering some form of cognitive therapy. Fifteen studies involved adult end users, three studies focused on 18–34-year-olds, three studies involved young people ranging between 12 and 17 years old, one study focused on adolescents aged 12–24-year-olds, and one study did not detail specific ages (but reported mean age = 21.4 years).

**Table 2.** Study characteristics.

	Author(s), year, country, study title	App name/ description of DMHI	Stakeholder(s)	Co-production phase (Discover, design, develop, test)	Co-production role	Co-production
1	Larkin et al. (2023)^39^, USAa ReachCare Mobile Apps for Patients Experiencing Suicidality in the Emergency Department: Development and Usability Testing Using Mixed Method	Emergency Department Safety Assessment and Follow up Evaluation (ED-SAFE) Safety planning for suicidality	*n = 27* *n* = 3 clinicians *n* = 4 ‘suicidologists’ *n* = 20 lived experience (interviews: 3 females, 6 males; 78% White, 11% Black or African American,11% other; testing: 2 females, 2 males; 1 non-binary; 80% White, 20% other)	Discover, design, develop and test (Discover -clinicians only)	Co-thinkers	Individual interviews, prototype testing and usability testing via a field user study Interviews with experts to understand patient's needs. Interviews with end users using design mock ups with users Tested the design efficacy and usability of apps in a field user study
2	Almeqbaali et al. (2022)^40^, UAEb A Biofeedback-Based Mobile App With Serious Games for Young Adults With Anxiety in the United Arab Emirates: Development and Usability Study	Biofeedback-based app with serious games for young adults with anxiety (i.e., relaxation techniques, mood tracking)	*n = unclear* unknown quantity of 18–37-year-olds for survey *n* = 2 mental health professionals *n* = 6 university students (18–25 years old) self-identified with anxiety *n* = 5 ‘experts’	Discover, design, develop and test (Testing - experts only)	Co-thinkers	Design and usability testing via survey, review of literature, semi-structured interviews, and usability prototype evaluation Phase 1: design and development of the app, involving i) a survey to investigate preferences for mobile games for stress and anxiety relief, ii) an analysis of serious games for anxiety iii) semi structured interviews with two mental health professionals, and six university students Phase 2: usability evaluation with experts testing the usability of the developed app
3	Atif et al.(2022)^41^, Pakistan Technology-assisted peer therapy: a new way of delivering evidence-based psychological interventions	An adaption of the THPc, a cognitive therapy-based intervention for perinatal depression	*n = 18* *n* = 6 expert group (mental health practitioners and developers of THPc) *n* = 10 user group (*n* = 4 service users (females), *n* = 2 partners, *n* = 4 health workers) *n* = 2 technology group (software developer and graphic designer)	Discover, design, develop and test	Partners	Qualitative work, desk-review, storyboard, cognitive walk through, user consultation and ‘think-aloud’ methods Phase 1: Qualitative work and a desk review to gather user needs and develop a storyboard. Storyboard reviews with user group. Phase 2: Usability laboratory testing with design team with a paper prototype using ‘cognitive walkthrough’ Phase 3: Usability field testing with end users using an advanced protype with ‘think aloud’ methods
4	Brannelly et al.(2022)^42^, New Zealand Co-production of digital mental health technologies to support individuals in mental health crisis	Self- management crisis recovery app	*n = 5* *n* = 4 service users *n* = 1 app developer	Discover, design, and develop	Partners	Consultation meetings Exploring what aspects people wanted in the app, developing the app, including a prototype.
5	Li et al. (2022)^43^, Australia A cognitive behavioural therapy smartphone app for adolescent depression and anxiety: co-design of ClearlyMe	ClearlyMe: CBTd app to target depression and anxiety in adolescents	*n = 38* *n* = 36 adolescents aged 12–17 *n* = 15 parents *n* = 32 mental health professionals	Discover, develop, design and test	Partners	Focus groups, workshops, consultations, prototype laboratory testing, interviews and ‘think aloud’ methods 1) Focus groups to discover users’ needs, views and preferences 2) Workshops and consultations to define app features 3) Designing CBTd content and visual features 4) Testing prototypes via interviews and using ‘think aloud’ methods
6	Alqahtani et al. (2021)^44^, Canada Co-designing a Mobile App to Improve Mental Health and Well-Being: Focus group study	Generating an app to improve mental health and well-being	*n* = 32 with mental health difficulties, age range = 18–34 years (16 males, 16 females)	Discover and design	Co-thinkers	Focus groups Six focus groups across three phases: 1) explore users’ experiences; 2) understand needs in relation to two mental health apps; 3) co-design session to design apps
7	Callan et al. (2021)^45^, USAa CBT Mobilework©: user-centered development and testing of a mobile mental health application for depression	CBTd skills app for depression	*n = 33* Phase 1, development: *n* = 8 depressed patients (mean age = 38.25 years; 87.5% females, 100% Caucasian) *n* = 5 therapists Phase 2, testing: *n* = 15 depressed patients (mean age = 41.87 years; 73.3% females, 80% Caucasian, 13% African American, 7% American Indian) *n* = 5 therapists	Develop, design and test	Co-thinkers	Usability testing Phase 1: refinement and iterative usability testing of the CBTd mobilework© prototype Phase 2: real world testing of CBTd mobilework©
8	Easton et al. (2021)^46^, England Blending cognitive analytic therapy with a digital support tool: Mixed methods study involving a user-centered design of a prototype app	CATe-app: to support engagement in the “recognition” phase of treatment	*n = 56* Survey: *n* = 50 CATe therapists Prototype user testing: with *n* = 3 CATe therapists (female) *n* = 3 ex CATe patients (2 females, 1 male)	Discover (only therapists), design and test	Co-thinkers	Online survey, prototype testing via cognitive walk through National survey to determine readiness to adopt apps in practice and identify content. Face-to-face testing of the prototype system via cognitive walk-through methods
9	Jonathan et al. (2021)^47^, USAa A Smartphone-Based Self-management Intervention for Bipolar Disorder (LiveWell): User-Centered Development Approach	LiveWell: self-management intervention for bipolar disorder; daily monitoring	*n* = 11 service users with bipolar disorder, (age range = 21–65 years, mean age = 36 years; 4 males, 7 females; 11 non-Hispanic white)	Design, develop and test	Co-thinkers	Design sessions via interviews, ‘think aloud’ technique and questionnaires, and usability field testing Design sessions: Think aloud sessions using a smartphone app mock up Usability testing sessions: field testing prototypes followed by interviews around general impressions, discussing possible scenarios and a questionnaire on overall usability
10	Patoz et al. (2021)^48^, France Patient and physician perspectives of a smartphone application for depression: a qualitative study	App targeting major depressive episode	*n = 50* *n* = 26 physicians (13 males, 13 females; mean age = 45.4 years) *n* = 24 patients (13 males, 11 females; mean age =51.5 years)	Discover and design	Co-thinkers	Review of literature, survey, focus groups and interviews Literature search on apps for depression to develop semi-structured interview guides. Surveys around app use habits. Perceptions and expectations around a hypothetical app were investigated via focus groups using semi-structured interviews with patients and physicians.
11	Berry et al. (2020)^49^, England Developing a Theory-Informed Smartphone App for Early Psychosis: Learning Points From a Multidisciplinary Collaboration	Actissist: Cognitive behaviour therapy intervention for early psychosis	*n = 106* Phase 1: *n* = 26 (16–34 years) service users with psychosis (mean age = 26 years; 11 females, 10 males) Phase 2: *n* = 48 (19–59 years) clinicians (mean age = 39.2 years; 27 females, 20 males; 1 missing data; white British = 40, mixed = 4, white Irish = 1 missing data = 3) Phase 3: *n* = 27 (16–35 years) service users with psychosis (mean age = 27 tears; 3 females, 11 males) *n* = 3 software engineers, *n* = 2 academics	Discover, design, develop and test	Partners	Expert reference groups, beta-testing, qualitative interviews, and focus groups Phase 1: Qualitative interviews with service users and focus groups with clinicians from early intervention services Phase 2: App prototype was beta-tested with mental health professionals and service users for feedback on functioning, design, and content Phase 3: Qualitative interviews about experience of app (those who had participated in proof-of-concept study)
12	Newton et al. (2020)^50^, Canada A Mobile Phone-Based App for Use during Cognitive Behavioural Therapy for Adolescents with Anxiety (MindClimb): User-Centred Design and Usability Study	MindClimb: CBTd app for young people with anxiety	*n = 42* Phase 1: *n* = 6 Youths (12–24 years); *n* = 3 trained CBTd clinicians Phase 2: *n* = 17 adolescents in treatment for anxiety, mean age = 15; *n* = 5 app developers Phase 3: *n* = 8 adolescents and 3 therapists	Discover, design, develop and test	Partners	Consultations, focus groups, think-aloud activities during usability testing cycles and interviews Phase 1: Predesign consultations via wireframing with young people and clinicians, which resulted in a low-fidelity MindClimb Prototype. Focus groups on low-fidelity prototype to develop a high-fidelity prototype. Phase 2: High fidelity prototype iterative cycles (acceptability, learnability and usability) testing via think aloud and gathering. Usability evaluation by app developers. Phase 3: Case series to assess usability (beta testing) among adolescents and therapists during group CBTd. Interviews around experience.
13	O’Grady et al. (2020)^51^, Ireland A Mobile Health Approach for improving Outcomes in Suicide Prevention (SafePlan)	SafePlan: suicide prevention, including a diary component facilitating the generalisation of skills from DBTe	*n* = 11 clinicians, psychologists, and information technology specialists *n* = 18 students (14–16 years)	Discover, design, develop and test (Testing by design team and students)	Partners	Review of existing apps, agile methodology, survey, design group meetings, design group workshops, focus groups using wireframe mock-ups and beta, and field testing. A review of existing mobile apps (*n* = 5), survey circulated to frontline professionals to assess feature preferences, which formed the basis of initial app design. Design group iterative cycle workshop with a variety of professionals. Further clinician survey. Beta testing by the core design team, and field testing by students.
14	Burchet et al. (2019)^52^, Germany/Sweden/Egypt User centered app adaption of a low intensity E-Mental health intervention for Syrian refugees	Adaption of Step-by-Step web-based intervention developed by the Interviews and focus groups	*n = 128 adult Syrian refugees* (Phase 1: *n* = 60, mean age = 33 years; phase 2: *n* = 36, mean age = 33.8 years; phase 3: *n* = 32, 16 males, 16 females)	Discover, design, and test	Co-thinkers	Interviews, focus groups, ‘think aloud’ methods, prototype testing Three phases: 1) Free list interviewing around technology; 2) key informant interviewing using ‘think aloud’ methods using an online prototype; and 3) focus group discussions
15	Christie et al. (2019)^53^, New Zealand Gamifying CBT to deliver emotional health treatment to young people on smartphones	Quest-Te Whitianga, a gamifying CBTd skills app for young people with emotional difficulties	*n = 88* whānau groups (*n* = 9), school health council group (*n* = 9), community youth group (*n* = 9), Pacific workshop (*n* = 11), Māori workshop (*n* = 20) Consultation with clinicians, focus groups (school and community) and interactive workshops with youth, *n* = 30 young people, aged 12–15 years	Discover, design, and develop	Partners	Consultations, review of literature, interviews, focus groups and interactive user workshops. Scoping phase: Interviews, focus groups and workshops from a range of stakeholders. Design and develop phase: ‘Sprints’ (short fixed-length time slots) with software company around app design and user experience combined with feedback from young people.
16	Derks et al. (2019)^54^, Netherlands Development of an Ambulatory Biofeedback App to Enhance Emotional Awareness in Patients with Borderline Personality Disorder: Multicycle Usability Testing Study	Sense-IT: Biofeedback app to enhance emotional awareness in patients with borderline personality disorder	*n* = 12 *n* = 5 service users (borderline personality disorder), mean age = 28 years, all females, for cycle 1 *n* = 4 health care professionals (1 psychiatrist, 1 bodily orientated psychotherapist, 2 group workers or sociotherapists), aged between 47–62 years, mean age = 52 years, for cycle 2 *n* = 3 expert users (within technology field) for cycle 3	Test	Co-thinkers	Questionnaires, semi-structured interviews, cycle testing, task scenarios and cognitive walk through Three cycles of testing: Cycle 1: Prototype testing with patients followed by questionnaires on the patient's level of experience with technology. Also, semi-structured interviews about the app. Cycle 2: Usability testing with professionals, involving task scenarios and interviews. Cycle 3: Usability testing with expert users via a cognitive walk through, by completing several tasks and asking to think aloud about what a primary user would do and evaluate how easy the task would be.
17	Hardy et al. (2018)^55^, England How Inclusive, User-Centered Design Research Can Improve Psychological Therapies for Psychosis: Development of SlowMo	SlowMo: based on the ‘Thinking well’ psychosis app, targeting reasoning styles that contribute to paranoia	*n = 18 adult service users with a diagnosis of nonaffective psychosis* (9 men, 9 females; 23–62 years of age; white British = 39%, black Caribbean = 17%, black African = 11%, black British = 11%, white British and black Caribbean = 11%, white British and black African =6%, white British and black Caribbean =6%)	Discover, develop, design and test	Partners	Case series and design research via interviews, observations of therapists, workshops, and prototype testing 1) Discover: case series of thinking well stakeholder interviews, desk research, user profiling, system mapping and a mood board 2) Define: workshops to generate design brief 3) Develop: concept workshops and prototype testing 4) Deliver: viable product was storyboarded
18	Hetrick et al. (2018)^56^, Australia Youth Codesign of a Mobile Phone App to Facilitate Self-Monitoring and Management of Mood Symptoms in Young People With Major Depression, Suicidal Ideation, and Self-Harm	Mood monitoring and management app for young people (CBTd and DBTf features)	*n = 27* *n* = 11 young people who had experienced depression, suicidal ideation (3 males and 8 females, mean age = 21.4 years) *n* = 6 clinicians (Clinical Psychologists, Social Workers, Psychiatrists, Occupational Therapists)	Discover, design, and develop	Partners	Design studio methodology via workshops Four co-design workshops with young people and two focus groups with clinicians, sketching and presenting ideas, consolidating best ideas into a final design
19	McClelland and Fitzgerald (2018)^57^, England A participatory mobile application (app) development project with mental health service users and clinicians	Behaviour change app for psychosis	*n = 14* *n* = 8 clinicians *n* = 6 service users	Discover and design	Co-thinkers	Focus groups To discuss user needs and support the design, development, and content of a behavioural change app Phase 1: Discuss utility of an app to support development ‘mock up app’ with clinicians and service users Phase 2: Focus groups with service users and one with clinicians to discuss ‘mock up app,’ to support development of app prototype Stage 3: Development of mobile app prototype based on feedback from service users and clinicians
20	Switsers et al. (2018)^58^, Belgium Users’ Perspectives on mHealth Self-Management of Bipolar Disorder: Qualitative Focus Group Study	Self-management app for Bipolar	*n = 16 service users* (9 women, 7 men, with a diagnosis of bipolar disorder and undergoing treatment, over 18, mean age = 42 years)	Discover and design	Co-thinkers	Focus groups Seven focus groups conducted by trained clinicians with users. Session 1: 4 groups, context mapping to gather insights into the users’ needs regarding self-management in general and mHealthg self-management of bipolar specifically Session 2: cocreation to assess information about the users’ needs concerning the functionality and design of a self-management app, including designing, and drawing up an app
21	Terp et al. (2018)^59^, Denmark A smartphone app to foster power in the everyday management of living with schizophrenia Terp et al. (2017)^60^, Denmark Collaborating with Young Adults Diagnosed with Schizophrenia: A Participatory Design Study to Shape the Healthcare System Terp et al. (2016)^61^, Denmark A room for design: Through participatory design young adults with schizophrenia become strong collaborators	MindFrame: self-management app for schizophrenia for young adults (18–35-year-olds)	*n = 36* Terp et al.^59^: Testing: *n* = 13 service users (4 males, 9 females, mean age =24.8 years) Terp et al.^60^: *n* = 6 young adult service users with FEPh (aged 19–27 years, all ethnic Danes) Terp et al.^61^: *n* = 4 service users (3 female, 1 male; mean age = 24.8), *n* = 7 HCP'si, *n* = 3 software designers, *n* = 1 graphic designer, *n* −1 graphic recorder and *n* = 1 team leader	Discover, design, develop and test	Partners	Observations, interviews, and workshops using design artefacts Three phased approach: Phase 1: Identification of needs through users’ observations (*n* = 45 h) and interviews^60^ Phase 2: Design and development through workshops with users, HCP'si, a researcher, and software designers to collaboratively design resources to accommodate the needs. Design artefacts were used (storyboarding, card sorting, mock-ups and paper prototypes) were used throughout 10 workshops^61^ Phase 3: Pilot-testing of MindFrame and qualitative interviews^59^
22	Fortuna et al.(2017)^62^, Lebanon Adapting a Psychosocial Intervention for Smartphone Delivery to Middle-Aged and Older Adults with Serious Mental Illness	Integrated Illness Management and Recovery for serious mental illness, including psychoeducation, coping skills and relapse prevention	*n = 10 users/ older adults with serious mental illness (mean age = 55.3years; White = 90%)* *n* = unknown quantity of clinicians, peer specialists, physicians, and engineers	Develop and test	Co-thinkers	Usability test using ‘think aloud’ and surveys Usability test including two cycles using the ‘think aloud’ prototype. Participants also completed a survey on confidence using the app
23	Schlosser et al. (2016)^63^, USAa Feasibility of PRIME: A Cognitive Neuroscience-Informed mobile App Intervention to Enhance Motivated Behavior and Improve Quality of Life in Recent Onset Schizophrenia	Personalised Real-time Intervention for Motivational Enhancement (PRIME): A cognitive neuroscience app to enhance motivated behaviour and improve quality of life in recent onset schizophrenia	*n = 35* *n* = 15 key stakeholders, involving individuals with schizophrenia, family members, treatment providers and research experts. Feasibility and acceptability testing: *n* = 20 (mean age = 23 years; males = 17, females = 3; Caucasian = 6, Asian = 6; African American = 4; other = 4) with a diagnosis of schizophrenia spectrum disorder from an EISj	Discover, design, develop and test	Partners	Design workshops, interviews and testing Two design workshops and a series of in-depth 1:1 in-person interviews During initial design workshops, potential values that would improve quality of life were generated. Each feature of the app was evaluated using experiential strategies such as prototyping specific features and presenting potential paper mock ups. Feasibility and acceptability: first iteration 10 participants evaluated the initial feasibility and acceptability. The results were then used to inform the next iteration of PRIME to be tested in a RCTk, with 10 participants.
24	Ben-Zeev et al. (2013)^64^, USAa Development and Usability Testing of FOCUS: A smartphone system for self-management of schizophrenia	FOCUS: self-management of schizophrenia grounded in cognitive model of psychosis and the stress-vulnerability model of schizophrenia (includes psychosocial intervention- behavioural activation and psychoeducation)	*n = 924* Stage 1: *n* = 904 service users with schizophrenia or schizoaffective disorder; mean age = 47 years; African American = 61%, Caucasian = 38%, Hispanic = 5% *n* = 8 practitioners Stage 2: MDTl team (*n* = unknown) Stage 3: *n* = 12 service users with schizophrenia or Schizoaffective disorder; mean age = 45 years; 67% males; 75% African American, 8% Caucasian, 17% Hispanic	Discover, develop, design and test	Co-thinkers	Surveys, group discussions and laboratory useability testing Stage 1: Survey for individuals (*n* = 904) with SMIm about ownership and use of mobile technologies Stage 2: Survey and group discussion with eight practitioners across a range of specializations and service models (community- based treatment, rehab, etc.,) around how an mHealthg intervention could be of greatest utility to users Stage 3: Laboratory useability testing of 2 cycles

**Abbreviations:** a USA, United States of America; bUAE United Arab Emirates; cTHP, Thinking Health Programme); dCBT, Cognitive Behavioural Therapy; eCAT, Cognitive Analytical Therapy; fDBT, Dialectical Behavioural Therapy; gmHealth, Mobile Health; hFEP, First Episode Psychosis; iHCPs, Health Care Professionals; jEIT, Early Intervention Team; kRCT, Randomised Controlled Trial; l MDT, Multi-Disciplinary Team; mSMI, Serious Mental Illness*.*

### Methods and approaches to involvement

The evidence in relation to methods and approaches taken to co-produce DMHIs is summarised below and in further detail in Tables 2 and 3. Overall, only three studies^49,50,59^ endorsed all key principles identified in our quality appraisal tool. Furthermore, the identified barriers and facilitators around the co-production process are also described below.

**Table 3.** Quality appraisal.

	**Design**		**Stakeholders**			**Aims**	**Phase**		**Inclusivity and power**		**Monitoring**
**Author(s), publication year**	1. Was there reference to any co-production guidelines or frameworks that were considered?	2. Was there reference to user-centred design methodology?	3. Were recruitment methods clearly outlined for co-production?	4. Were attempts made to ensure participation was representative of the user population for the DMHI?	5. Was the ‘end user’ involved in the co-production process?	6. Were the intended outcomes of stakeholder involvement clearly outlined?	7. Was co-production embedded from the beginning of the research process?	8. Were end users involved in the co-production throughout all stages of the research? 1) discover 2) design 3) develop and 3) testing	9. Was consideration given to addressing and minimising power imbalances across stakeholders (i.e., shared decision making)?	10. Were efforts made to ensure co-production activities were inclusive and accessible?	11. Was the impact of co-production on design and outcomes clearly monitored and outlined, i.e., were changes made clearly based on feedback?
Larkin et al.(2023)^39^	Yes	Yes	No	Yes	Yes	Yes	Yes	No	Unclear	Unclear	Yes
Almeqbaali et al.^40^ (2022)	No	Yes	Yes	Yes	Yes	Yes	Yes	No	No	Unclear	Yes
Atif et al. (2022)^41^	No	Yes	No	Yes	Yes	Yes	Yes	Yes	Yes	Yes	Yes
Brannelly et al. (2022)^42^	No	Yes	No	Yes	Yes	Yes	Yes	No	Yes	Unclear	Yes
Li et al. (2022)^43^	Yes	Yes	Yes	Yes	Yes	Yes	Yes	Yes	Yes	Unclear	Yes
Alqahtani et al. (2021)^44^	No	Yes	Yes	Yes	Yes	Yes	Yes	No	Unclear	Unclear	Yes
Callan et al. (2021)^45^	No	Yes	No	Unclear	Yes	Yes	No	No	Unclear	Unclear	Yes
Easton et al. (2021)^46^	Yes	Yes	Yes	Yes	Yes	Yes	No	No	Unclear	Unclear	Yes
Jonathan et al. (2021)^47^	No	Yes	Yes	Yes	Yes	Yes	No	No	Yes	Unclear	Yes
Patoz et al. (2021)^48^	No	Yes	Yes	Yes	Yes	Yes	Yes	No	Unclear	Unclear	Unclear
Berry et al. (2020)^49^	Yes	Yes	Yes	Yes	Yes	Yes	Yes	Yes	Yes	Yes	Yes
Newton et al. (2020)^50^	Yes	Yes	Yes	Yes	Yes	Yes	Yes	Yes	Yes	Yes	Yes
O’Grady et al. (2020)^51^	No	Yes	No	No	No	Yes	Yes	Yes	Yes	Unclear	Yes
Burchet et al. (2019)^52^	Yes	Yes	Yes	No	No	Yes	Yes	No	Unclear	Unclear	Yes
Christie et al. (2019)^53^	No	Yes	No	Unclear	Yes	Yes	Yes	No	Unclear	Unclear	Yes
Hardy et al. (2018)^55^	Yes	Yes	Yes	Yes	Yes	Yes	Yes	Yes	Yes	Yes	Yes
Hetrick et al. (2018)^56^	Yes	Yes	Yes	Yes	Yes	Yes	Yes	No	Yes	Unclear	Yes
McClelland & Fitzgerald (2018)^57^	No	Yes	Yes	Yes	Yes	Yes	Yes	No	Yes	Unclear	Yes
Switsers et al. (2018)^58^	No	Yes	Unclear	Yes	Yes	Yes	Yes	No	Unclear	Unclear	Unclear
Terps et al. (2018; 2017; 2016)^61^06/10/2023 23:41:0006/10/2023 23:41:00	Yes	Yes	Yes	Yes	Yes	Yes	Yes	Yes	Yes	Yes	Yes
Derks et al. (2019)^54^	Yes	Yes	Yes	Yes	Yes	Yes	No	No	Yes	Yes	Yes
Fortuna et al. (2017)^62^	No	Yes	No	Yes	Yes	Yes	No	No	Unclear	Yes	Yes
Schlosser et al. (2016)^63^	No	Yes	No	Yes	Yes	Yes	Yes	Yes	Yes	Unclear	Yes
Ben-Zeev et al. (2013)^64^	No	Yes	No	Yes	Yes	Yes	Yes	Yes	Unclear	Unclear	Yes

### User involvement

Across all studies reviewed, between 5 and 924 stakeholders were involved in the co-production process, with a combined reported sample of 1768. Type of participants and stakeholders included in the co-production process varied, but representatives from the target population (‘end users’) were included in most studies (*n* = 21), apart from three.^43,51,52^ Two studies explicitly chose not to include population representatives due to the perceived sensitivity of the target population (e.g. suicidal ideation^51^; e.g. refugees).^52^ For example, O’Grady et al.^52^ used age-matched controls instead of young people with suicidal thoughts; although, this was recognised as a limitation. Burchet et al.^53^ included 128 Syrian refugees, however they were not pre-screened for mental health difficulties, and therefore recognised they did not necessarily represent the DMHIs target group. Additionally, Li et al.^44^ opted against restrictive recruitment to capture a broad range of user views, including those who did not self-identify as having mental health difficulties. Other stakeholders involved in co-producing DMHIs included: clinicians (17 studies); technological or app experts (6 studies); members of the public (5 studies); family members (2 studies); and academics/researchers (3 studies). Information on demographics, particularly gender and ethnicity of all stakeholders was generally poorly reported. Gender was reported by 14 studies, 11 studies reported age, and eight studies reported ethnicity. Two studies did not report any demographics.^43,58^

### Co-production phase

Nineteen of the 24 studies reported co-producing the DMHI from the outset, which aligns with the first stage of ‘discover’ within the double diamond design framework for developing and evaluating interventions. Twenty-two studies involved stakeholders in designing the DMHI and 17 within the development stage. The final stage of the reported co-production process, prototype testing, was reported in 14 studies. However, an additional three studies did report testing the DMHI, but with ‘experts’,^41^ a design team and students^52^ and a sample that may not represent the target population.^53^ Additionally, less than half of the studies (*n* = 9) co-produced throughout all four stages of involvement (discover, design, develop and test).

### The role of involvement

Whilst all studies made explicit reference to the intended outcomes of stakeholder involvement and utilising co-production methodology, less than half of the studies (*n* = 10) referred to co-production guidelines. The Involvement Matrix tool^30^ to distinguish the five roles of co-production involvement (listener, co-thinker, advisor, partner, and decision-maker) was used to determine the roles of stakeholders in the included studies (see Table 2). In 13 of the studies, stakeholders were classified as having the role of ‘co-thinkers’, which involves providing opinions throughout each stage. For example, Fortuna et al.^63^ conducted a usability test with five end users and developed a second version of the app following user feedback. In 11 studies, stakeholders were classified as ‘partners’ involved as shared decision makers. For example, in Hardy et al.'s^56^ ‘discover’ phase, all stakeholders (service users, carers, therapists and clinicians) developed a shared understanding of psychological therapy, behaviour change, psychosis and technology use from the perspective of various stakeholders, to develop the most intuitive ways of communicating these ideas. The highest form of involvement within the matrix, the ‘decision-maker’ role, where end users take initiative and/or make final decisions, was not identified in any studies.

### Methods of co-production

The methods used to facilitate the co-production process are displayed in Table 4. Methods included more traditional design methodology: interviews (*n* = 13); focus groups or group discussions (*n* = 17); surveys or questionnaires (*n* = 7); consultations (*n* = 5); reviewing literature (*n* = 5) and observations (*n* = 2). More unique methods of co-production involved ‘think aloud’ or cognitive walk-through methods^65^ in eight studies. These methods examine the usability of a product, where stakeholders are asked to carry out tasks while thinking out loud about what they would do and evaluate whether the task at hand is easily achievable. For example, Derks et al.^55^ asked end users and therapists to complete tasks whilst asking, “Will the primary user notice that progress is being made toward accomplishment of their goal?” (p. 7). Other methods including creative methods using design artefacts (*n* = 8), such as storyboarding, card sorting, mock-ups drawings and paper prototypes.

**Table 4.** Methods used for co-producing.

		Methods										
	Author(s) and publication year	Focus groups/group discussions	Consultations	Semi- structured interviews	Surveys/ questionnaires	Prototype testing Real-world Lab Field Beta			“Think aloud”/ cognitive walk though	Review of resources/literature	Design artefacts*	Observations
1	Larkin et al. (2023)^40^			X		X						
2	Almeqbaali et al. (2022)^41^			X		X				X		
3	Atif et al. (2022)^42^	X	X			X		X	X	X	X	
4	Brannelly et al. (2022)^43^		X									
5	Li et al. (2022)^44^	X	X	X		X			X			
6	Alqahtani et al. (2021)^45^	X										
7	Callan et al. (2021)^46^					X		X				
8	Easton et al. (2021)^47^				X	X			X			
9	Jonathan et al. (2021)^48^	X		X	X		X		X			
10	Patoz et al. (2021)^49^	X		X	X					X		
11	Berry et al. (2020)^66^	X		X				X				
12	Newton et al. (2020)^51^	X	X	X		X		X	X		X	
13	O’Grady et al. (2020)^52^	X			X	X		X		X		
14	Burchet et al. (2019)^53^	X		X		X			X			
15	Christie et al. (2019)^54^	X	X	X						X		
16	Derks et al. (2019)^55^			X	X	X	X	X	X			
17	Hardy et al. (2018)^56^	X		X		X					X	X
18	Hetrick et al. (2018)^57^	X									X	
19	McClelland & Fitzgerald (2018)^58^	X									X	
20	Switsers et al. (2018)^62^	X									X	
21	Terps et al.^59^	X		X		X		X			X	X
22	Fortuna et al. (2017)^63^				X	X			X			
23	Schlosser et al. (2016)^64^	X		X		X		X			X	
24	Ben-Zeev et al. (2013)^75^	X			X	X						

*Storyboarding, card sorting, drawings mock-ups, wire framing and paper prototypes.

App prototype testing, which involves a preliminary visual mock-up that looks like a real app and demonstrates an app's fundamental design and function, was conducted in seventeen studies. App prototypes can be as basic as sketches, or as high-fidelity as a clickable, digital model that works on stakeholders’ phones. Prototype testing is believed to be the most effective way of knowing how a product will perform. The final version of apps were tested by stakeholders in laboratory settings (*n* = 15), or real-world setting (*n* = 10). Real world testing was achieved through either field testing (*n* = 3), evaluating the adoption of product features, where stakeholders roam freely by exploring any exhibit they choose to test usability rather than content; or, through beta testing (*n* = 8), which aims to evaluate satisfaction and ensure release readiness. Beta testing involves a focused tour, where certain functions are directly presented to the user and evaluated. Prototype testing was often combined with other methods. For example, Burchet et al.^53^ used ‘think aloud’ methods based on initial impressions and feedback, while stakeholders engaged with the prototype app, asking, ‘Do you think that this app can be helpful for Syrian refugees here in [country] who experience sadness or distress?’ (p. 5).

### Challenges and added value to co-producing DMHIs

Co-production with stakeholders yielded various advantages and obstacles for the development and implementation of DMHIs.

### Challenges to co-production

#### Resource constraints

One of the main challenges encountered in the co-production process was practical constraints. Several studies^43,47,51,53,58,66^ reported issues related to resources, time and costs that constrained co-production activities. Researchers recognised resource constraints restricted them from being able to always explore and address all stakeholder suggestions. Time restraints meant that Easton et al.^47^ were unable to explore suggestions around creative approaches to engaging people in therapy as they prioritised reviewing app content.

Practical challenges were exacerbated by the digital nature of the intervention development and testing. Some stakeholders did not have access to mobile phones and could therefore not test the tool.^40,46,53^ In other cases, the expense involved in iterating and creating multiple versions of the app was prohibitive,^51,53,58,66^ and technical limitations in incorporating user feedback into app features was also a challenge.^48,50^ For example, in Berry et al.,^66^ end users requested multi-media features, such as voice recording features, that were not feasible for the software team to develop due to time and finding constraints, limited resources within the study time frame or the funding of the research grant. Moreover, where researchers asked stakeholders to test a prototype version of the app that did not necessarily reflect the end-product,^53^ stakeholders reported feeling less engaged in, and satisfied with, the co-production process. Furthermore, some studies noted that, in some instances, stakeholders changed their views or preferences over time, or hypothetical ideas did not always translate well into practice, resulting in the requirement of further changes and increased costs. For example, two studies reported that initial preferences identified by stakeholders in the ‘discovery stage’ were not endorsed during subsequent stages (e.g. Chatbot features^44^; number of alert notifications^66^).

#### Recruitment challenges and commitment

A common barrier reported in most studies reviewed^40,47,51,54,55,59,61,62^ was the use of small samples to co-produce, reducing generalisability and influencing the nature of the input received. Several studies further noted the lack of recruiting a diverse sample as a challenge,^39,40,42–45,51,53^ and consequently queried how representative the views were to the wider target audience the DMHI was designed to help. Studies recognised methods used to recruit influenced the limited diversity of the sample, such as recruiting from services, where individuals may be more help-seeking and/or have a better self-management of their mental health.^57,58^ Researchers also acknowledged online recruitment was likely to target more proficient users of technology, thus leading to a more biased sample in terms of familiarity with, and openness to, smartphones.^44,47^

Another challenge related to recruitment was around managing risks (i.e. suicidal ideation) within groups of more vulnerable users.^51,53,57^ Researchers recognised it was important to ensure the safety and well-being of end users in the event they found taking part in co-production work distressing, especially when this involved sharing personal experiences. As indicated previously, the concern regarding managing safety led to one study excluding end users^52^ and is at odds of the fundamentals of co-production.^26^ Furthermore, as Burchet et al.^53^ recruited Syrian refugees, audio recordings of the interviews were not made due to privacy concerns raised by the sample. Instead, a written record was made, and researchers recognised their approach to data collection is likely to have affected data quality and depth.

Research teams also had to adapt to the changing circumstances of stakeholders, such as rescheduling meetings due to low attendance.^58,66^ Berry et al.^66^ described how inconsistency with user attendance meant end users were unable to take a leader role to co-chair meetings, and co-production relationships were harder to build.

#### Conflicting views and expectations

An important aspect of effective co-production was achieving a balance between stakeholders’ suggestions, whilst acknowledging the diversity of their needs and preferences. Several studies^43–45,47,53,62,66^ noted variability in views and opinions expressed about topics covered in co-production sessions, which led to difficult decisions needing to be made by the research team around how to integrate and reconcile suggestions into the next iteration of app development. For example, Branelley et al.^43^ described stakeholders expressing conflicting requests for alert notifications via the app: some stakeholders found alert notifications helpful, while others found these intrusive. Preferences also varied depending on how stakeholders intended to the use the app, with some expressing the need for immediate crisis support as an important feature of the app, and others preferred prioritisation of longer-term features, such as tracking.^45^ Moreover, two studies^47,66^ reported that some user, and even clinician suggestions were at odds with the theoretical underpinnings of the intervention.

Additionally, some studies^44,47,66^ encountered challenges in accommodating the preferences across different stakeholder groups. For instance, when Li et al.^44^ co-produced a smartphone app for young people with anxiety and depression, parents valued credibility, professionals valued safety, and young people desired accessibility, relatability, and reliance. Other studies identified they were not always able to capture the views of all stakeholder groups, including engaging family members due to recruitment and practical issues, limiting experiences across stakeholder groups.^40^

#### Power imbalance

A further barrier included power imbalances between stakeholders and researchers’ methodological approaches, including focus groups were described as a contributing factor to an imbalance in power. Some studies felt end users did not voice ideas or talk about personal experiences due to group dynamics.^47,49,53,54,66^

Studies generally did not report on inclusivity, power, and equality in decision making within co-production processes, which are key principles within the NIHR INVOLVE^26^ guidance; with only seven of the 24 studies reporting on any efforts made to ensure the inclusivity and accessibility of co-production activities. Furthermore, ratings on the methodological quality tool (Table 3) demonstrated only thirteen studies reported on attempts to minimise power imbalances. An example of high-quality approaches to inclusivity included Terp et al.'s^39^ study who explicitly described how they supported a young adult user with low writing and literacy skills by putting their story into words, and two end users who lacked the confidence to present their ideas to the wider group were supported by team leaders. However, nearly all the studies (*n* = 22) reported on changes to interventions based on stakeholder feedback, using specific examples. For example, Atif et al.^42^ found end users preferred brief messages due to low literacy and effects of depression, therefore the content was broken down into small segments with just one or two key messages, to meet the needs of end users.

### Added value

#### Enhancing cultural sensitivity

The included studies identified the co-production of DMHIs delivered through apps enabled researchers to be culturally sensitive when developing the intervention through gathering feedback around language and relatable examples.^41,42,53,54,58^ Researchers described how the inclusion of stakeholders from diverse (ethnic, cultural, age and gender) backgrounds allowed the development of a DMHI that resonated with and reflected the wider target populations. Such alignment of the app with the end users’ language, cultural and context enhanced the relevance, acceptability, and usability of the app.^41,53,54^

#### Enrichment of ideas

The diversity of stakeholders enriched the variety of ideas and views beyond the researcher's perspective.^49,56,66^ Indeed, Berry et al.^66^ reported that some features included in the Actissist app would not have been considered by the research group without multiple stakeholder input, such as recovery videos. Co-production activities ensured that a range of stakeholder views and needs captured key information and skills that could be integrated into DMHI development, ensuring a more creative and innovate app could be developed.^41,57^

#### Acceptability and usability

Co-producing DMHIs with stakeholders facilitated the development of useful, relevant, and acceptable apps that met both the end users’ and researchers’ needs and concerns.^40,45,51,58,60^ Specifically, Newton and colleagues^51^ described how the co-production process resulted in an app that felt relevant and acceptable to both clinicians and service users, showing promise in exposure interventions outside of therapy. Several studies also emphasised the necessity of iterative testing, to identify and incorporate the needs and features identified and prioritised by stakeholders, such as design and content flaws noted in prototypes, in a timely manner.^46,53,64^ For instance, one study found that after end users engaged in laboratory prototype testing of an initial version, larger visual aids were required for their population of older people.^63^ Adapting the app with larger visual aids improved the appearance and navigation functions of the app, thus increasing its usability and acceptability. The co-production process also afforded the research team insight into which content and design features were/were not acceptable, and generated concrete recommendations for improvement before the app went live, such as simplifying information,^41^ modifying terminology^53^ and adding goal setting functions.^66^ Therefore, reviewing content with stakeholders early in the design process enabled refinement of content that balanced usability and informativeness, in line with the evidence base. Stakeholders also reported co-production methods increased their trust in the DMHI being developed.^49^

#### Methods promoting engagement

Certain methodology, including creative methods, such as ‘think aloud’ interviews were felt, by researchers, to improve engagement and increase honest feedback.^39,53,54^ Christie et al.^54^ changed their methodological approach after noticing a reluctance for end users to express views during interviews and focus groups. However, the employment of ‘think aloud’ methods, whereby the young person immersed themselves in the app and then gave one-to-one feedback in response to more targeted questions, facilitated engagement,^54^ and researchers felt feedback was more authentic and meaningful. Terp et al.^39^ also described creative workshops using several design artefact techniques (storyboard, card sorting, mock-ups and paper prototypes) supported active participation, specifically the use of card sorting, where stakeholders arranged ‘problem statements’ into categories, which supported the position preferences for the content of the app.

#### A sense of community

A further reported benefit of co-producing smartphone apps was the opportunity to collaborate with stakeholders as part of a community, with mutual engagement where stakeholders’ voices felt heard. Stakeholders appreciated the facilitation of connections with others,^43,60^ the exchange of ideas^49,52,56^ and the enthusiasm it generated.^39,45,47,57^ For example, end users described feeling seen as knowledgeable resources instead of a patient in need of care.^39^

## Discussion

The objective of this review was to provide an overview of the methods, and approaches to co-producing mental health app interventions, alongside the barriers and facilitators to co-production. This study represents, to the best of our knowledge, the first systematic review to assimilate the literature and provide a robust summary of the co-production activities of DMHIs via apps. This review highlights the existence of notable gaps of evidence concerning co-produced DMHIs. Specifically, there is significant variability in terms of stakeholder groups involved, stage and role of involvement, methods used, consideration of frameworks, and attempts to minimise power dynamics. The review identified four distinct yet overlapping concepts pertaining to the barriers of successful co-production of DMHIs: (a) resource constraints; (b) recruitment challenges; (c) conflicting views; and (d) power imbalances. The added value of co-producing DMHIs was identified as: (a) enhancing cultural sensitivity; (b) enrichment of ideas; (c) increased acceptance of DMHI; (d) methodology promoting engagement; and (e) a sense of community.

A key finding was the limited reporting of stakeholder demographics, co-production frameworks, attempts to ensure inclusivity, attempts to minimise power dynamics, and the equality in decision making. This is a common criticism within the research literature where authors rarely detail the activities taken to align with the key principles of co-production.^67^ Possible reasons for the limited reporting could be due to the absence of a uniformed co-production framework to apply co-production in practice. Alternatively, this may be explained by publication word limits, especially given that most studies’ objectives were around the co-produced intervention content and rarely primarily aimed at reporting the co-production process. The findings of this review call for researchers to report co-production activities to allow for co-production principles to be better operationalised in practice.

The value of involving stakeholders throughout all phases is well documented for producing relevant and useful DMHIs for people experiencing mental health difficulties. Most studies (*n* = 23) involved end users at a ‘co-thinker’ level, and sometimes as ‘partners’, but never ‘decision makers’, suggesting there is a still a need to improve equality between end users and researchers in the co-production process. Phase of involvement was variable, with highest user involvement during the first stage of the co-production process, and only nine studies involving end users throughout all stages of co-production. The findings from this review overlap with the systematic review conducted by Veldmeijer et al.,^31^ who explored the extent to which stakeholders were involved in mental healthcare through design, including DMHIs. Both reviews found that the level of involvement of end users was lower in the latter phases. This finding aligns with the wider healthcare co-production literature where co-production involvement tends to be limited to the early stage, with less involvement during the middle ‘design’ phase.^24,68^

According to Sanders et al.,^16^ active participation is a well-known challenge to successful co-production. A novel finding of this review was that certain methodological approaches such as ‘think-aloud’ techniques increased active participation and authentic engagement amongst individuals with mental health difficulties. Additionally, creative methods such as the utilisation of storyboarding alleviated the challenge of translating hypothetical ideas into practice. Therefore, more novel methodologies have the potential to improve co-production engagement^69^ and may also circumvent power issues and other difficult group dynamics that manifest in focus groups.

A dominate discourse in the wider literature of the challenges limiting co-production activity, are budget costs and time restrictions.^70^ This review found resources challenges were particularly prominent in the context of digital intervention due to the cost involved in their production. Building an app takes several months and has average related costs ranging from $60,000 to $250,000,^71^ with increased prototype developments increasing fees. Thus, adequate budgeting for prototypes, along with realistic time expectations to incorporate feedback, needs to be considered when co-producing apps. Prototype costs is of particular concern given the findings of this review suggest prototype versions of apps that are more closely reflective of the end-product are important to increase user engagement, and to ensure ideas translate into practice.

The included studies highlighted the need to recruit larger, heterogenous samples. A clear concern was the lack of diversity amongst stakeholders, and the co-production sample not being representative of the target audience. This concern is well documented across health research, whereby those involved in research are unrepresentative of the wider audience, especially ethnic minority groups.^72,73^ Whilst a larger, diverse sample may be more representative and encompass a wide range of ideas and preferences,^74^ it requires a process of careful negotiation for researchers to prioritise increased and varying ideas.^72^

There is significant value of co-production to maximise DMHIs acceptability and engagement through allowing end users voice's to be heard. Based on the good practice of co-production from the studies as evaluated through the bespoke quality tool, recommendations for co-producing DMHIs are made and presented in Table 5. Key highlights include the recruitment of a diverse population involving multiple stakeholder groups, involvement throughout all stages, steps taken to minimise power and promote equality in decision making, employment of think aloud and creative methods to facilitate participation and outlining non-negotiables of adaptions from the outset (i.e. ideas that interfere with the fidelity of the intervention).

**Table 5.** Clinical implications and recommendations.

Area to be considered	Recommendation
1. Careful consideration regarding sample: diversity, and stakeholder groups	Aim to recruit a diverse population, as cultural adaption of DMHIs has been identified as a factor that increases the effectiveness of DMHIs. Use multiple stakeholder groups to ensure a diverse range of views are captured (e.g., include parents when developing apps for adolescent populations).
2. The role and phase of involvement	Involve stakeholders throughout all stages of the co-production process, from the initial stage of ‘discover’ where ideas are generated, through to the implementation stage. Particular consideration should be given to the middle stage of ‘develop’ to ensure end-user voices are incorporated throughout every stage to develop an acceptable and useable DMHI.
3. Inclusivity and power	Proactively take steps to minimise power dynamics. For example, upon the set-up of the co-production group, acknowledge the power dynamic and make attempts to minimise the power differential by, for example, referring to members by names, not professional titles. Support end-users to lead with decision making. Co-production activities should be inclusive and accessible, such as adapting resources and sneering the equipment needed for meaningful involvement is supplied (e.g., tablet access if groups occur online).
4. Use of creative methods	Use collaborative ‘think aloud’ and other creative methods where possible, as traditional focus groups can inhibit participant responses, particularly in younger populations.
5. Process of negotiation	Consider feedback from all stakeholders, but balance this with fidelity to the evidence-based intervention in development. It might not be possible to meet all user group's needs, which could negatively influence co-production experiences. Outline the non-negotiables of the intervention in terms of theory and rationale at the outset of the co-production process. An open, honest, and sensitive approach should be taken to discuss why any changes were not possible, and where possible, compromises should be made as a collective. Guide negotiations in a way that manages expectations whilst fostering the relationship with stakeholders and minimising power dynamics.

The purpose of this review was to provide an overview of the methods and approaches to DMHIs. Therefore, broad search terms were used to include as many studies as possible that cover the topic and provide a complete overview. The number of included studies, the breadth of co-production methods and user involvement included in this review are a clear strength. A further strength is the development of a co-produced bespoke quality appraisal tool, and whilst the tool is not validated, it was able to capture a much richer understanding of the approaches to co-production.

Given that the search was limited to studies written or translated in the English language and those published in peer-reviewed journals, publication and language biases are acknowledged. Inclusion of other languages may have yielded more studies focused on low and middle-income countries. Nevertheless, a variety of studies conducted internationally were identified and included. It is important to note that included studies may be influenced by selection bias. Only studies that documented the co-production process were included. Thus, studies that have co-produced DMHIs, but did not report on the process, have not been captured. Therefore, the results need to be considered within this context, and this review may over-represent the co-production that is happening more widely.

## Conclusions

Findings suggest there is huge variation in terms of how co-production is being facilitated. Stakeholder involvement and the barriers and facilitators faced when co-producing DMHIs are emphasised. Based on the findings, important considerations for co-producing DMHIs are highlighted. Key recommendations for co-producing DMHIs include involvement of a diverse stakeholder group, involvement at all stages, inclusivity and roles to be considered, the balancing of suggestions and fidelity, and the inclusion of collaborative creative and ‘think aloud’ methods to allow for authentic feedback. Overall, a range of stakeholders, throughout the entirety of the design, development, and employment of DMHIs through various methodologies is advocated by this review to lead to evidence based and effective DMHIs for people with mental health difficulties.

Considering the insights gleaned from this review and the wider literature, it is imperative that forthcoming research directs its attention towards understanding stakeholder's perspectives of co-production. Additionally, investigation should be undertaken to evaluate the efficacy of implementing a co-produced framework across all four developmental phases of DMHIs. Consequently, empirical testing is warranted to ascertain whether DMHIs developed through co-production yield superior clinical outcome. Additionally, the co-produced bespoke tool used to assess co-production methodological quality should be used as a framework for researchers and app developers to implement co-production in adherence to the core principles.

## Supplemental Material

sj-docx-1-dhj-10.1177_20552076241239172 - Supplemental material for Co-producing digital mental health interventions: A systematic review Supplemental material, sj-docx-1-dhj-10.1177_20552076241239172 for Co-producing digital mental health interventions: A systematic review by Rebecca Brotherdale, Katherine Berry, Alison Branitsky and Sandra Bucci in DIGITAL HEALTH

sj-docx-2-dhj-10.1177_20552076241239172 - Supplemental material for Co-producing digital mental health interventions: A systematic review Supplemental material, sj-docx-2-dhj-10.1177_20552076241239172 for Co-producing digital mental health interventions: A systematic review by Rebecca Brotherdale, Katherine Berry, Alison Branitsky and Sandra Bucci in DIGITAL HEALTH
