# Supplementary material for: Co-producing digital mental health interventions: A systematic review
Source: Digit Health. 2024 Apr 25;10:20552076241239172. doi: 10.1177/20552076241239172 (PMC11044797; doi:10.1177/20552076241239172)
Supplement: sj-docx-1-dhj-10.1177_20552076241239172 - Supplemental material for Co-producing digital mental health interventions: A systematic review [file sj-docx-1-dhj-10.1177_20552076241239172.docx]

**Supplementary material 1. Bespoke methodological quality appraisal tool.**

**Bespoke quality appraisal tool for co-production**

**Eleven question co-production checklist. The questions can be answered, ‘yes’, ‘no’ or ‘unclear’.**

| Design | | | | | |
| --- | --- | --- | --- | --- | --- |
| 1. **Was there reference to a co-production framework/model that was considered?** | Yes  No  Unclear | | | *HINT:*  Consider a range of frameworks, such as, the National Institute for Health Research (NIHR) UK standards for Public Involvement | |
| *Comments:* | | | | | |
| 1. **Was there reference to user-centred design methodology?** | | Yes  No  Unclear | *HINT:*  Consider user-centred design methodology or similar | | |
| *Comments:* | | | | | |
| Stakeholders | | | | | |
| 1. **Were recruitment methods clearly outlined for co-production?** 2. **Were attempts made to ensure participation was representative of the user population for the DHI?** 3. **Was the ‘end user’ involved in the co-production process?** | | Yes  No  Unclear  Yes  No  Unclear  Yes  No  Unclear | | | *HINT:* Did the study recruit the right people to support co-production for that study? E.g., if the study focused on psychosis, was effort made to recruit a heterogeneous population of individuals diagnosed with psychosis  *HINT:* Was the stakeholder group of the intended user of the DHI involved in the co-production |
| *Comments:* | | | | | |
| Aims for co-production | | | | | |
| 1. **Were the intended outcomes of stakeholder involvement clearly outlined?** | Yes  No  Unclear | | | *HINT:*  Consider reference to any visions for PPI involvement. Is there reference to a shared understanding and agreement of the problem being tackled? | |
| *Comments:* | | | | | |
| Phase of involvement | | | | | |
| 1. **Was co-production embedded from the beginning of the research process?** 2. **Were end users involved in the co-production throughout all stages of the research?**   1) **Discover**; gathering user needs and preferences  2) **Design**; initial designing of the DHI and the content  3) **Development**; initial development of the DHI  4) **Testing**; testing the DHI | | Yes  No  Unclear  Yes  No  Unclear | | | *HINT:*  At what point did co-production happen? Were they involved in defining the issue/problem?  Were end users involved throughout the entire process from ideas through to testing? |
| *Comments:* | | | | | |
| Inclusivity and power | | | | | |
| 1. **Was consideration given to addressing and minimising power imbalances across stakeholders (i.e., shared decision making)** | | Yes  No  Unclear | *HINT:*  Were attempts made to acknowledge, address, and rectify power differentials between stakeholders? Is it clear that everyone is equally important/had an equal voice? | | |
| *Comments:* | | | | | |
| 1. **Were efforts made to ensure co-production activities were inclusive and accessible?** | | Yes  No  Unclear | *HINT:*  Ensuring accessibility for all; therefore, appropriate, and adequate adaptions (i.e., easy read documents -if required) were made? | | |
| *Comments:* | | | | | |
| Monitoring | | | | | |
| 1. **Was the impact of co-production on design and outcomes clearly monitored and outlined, i.e. were changes made clearly based on feedback?** | | Yes  No  Unclear | *HINT:*  Was progress reviewed? Was it clear that decisions/ changes were made based on PPI feedback? | | |
| Comments: | | | | | |
